# Direct Reading of *Bona Fide* Barcode Assays for Diagnostics with Smartphone Apps

**DOI:** 10.1038/srep11727

**Published:** 2015-06-30

**Authors:** Jessica X. H. Wong, Xiaochun Li, Frank S. F. Liu, Hua-Zhong Yu

**Affiliations:** 1 Department of Chemistry, Simon Fraser University, 8888 University Drive, Burnaby, British Columbia V5A 1S6, Canada; 2 Key Laboratory of Advanced Transducers and Intelligent Control Systems (Ministry of Education), College of Physics and Optoelectronic Engineering, Taiyuan University of Technology, Shanxi 030024, P.R. China

## Abstract

The desire to develop new point-of-care (POC) diagnostic tools has led to the adaptation of smartphones to tackle limitations in state-of-the-art instrumentation and centralized laboratory facilities. Today’s smartphones possess the computer-like ability to image and process data using mobile apps; barcode scanners are one such type of apps. We demonstrate herein that a diagnostic assay can be performed by patterning immunoassay strips in a *bona fide* barcode format such that after target binding and signal enhancement, the linear barcode can be read directly with a standard smartphone app. Quantitative analysis can then be performed based on the grayscale intensities with a customized mobile app. This novel diagnostic concept has been validated for a real-world application, i.e., the detection of human chorionic gonadotropin, a pregnancy hormone. With the possibility of multiplex detection, the barcode assay protocol promises to boost POC diagnosis research by the direct adaptation of mobile devices and apps.

Medical diagnostic tools (e.g., biosensors) respond to biochemical and physiological signals of interest and relay this information in the form of electrical or optical output, thereby reliably diagnose diseases and illnesses^1^. These devices, however, while readily accessible in developed countries, are often costly and complex to the average untrained person, and not easily accessible in all regions of the world^2^. To aid in reducing global health care costs, point-of-care (POC) diagnostics aims to provide patients with rapid real-time clinical testing and treatment, primarily in developing countries which are lack of medical facilities. Diagnosis systems of this caliber must be inexpensive, easy to use and disposable due to limitations in trained personnel, infrastructure, medical instruments, and operational funds^3^,^4^. Recent diagnostics research is showing a trend toward not only the design of low-cost instruments that are easy to use^5^,^6^,^7^,^8^,^9^,^10^,^11^,^12^,^13^, but also the integration of testing applications into mobile devices. Smartphones, which have millions of users worldwide including third-world regions, can be adapted for diseases detection^2^,^3^,^4^, and the wireless system can relay patient information and test results to health professionals for further analysis if necessary. To date, POC devices already in use include glucose meters, pregnancy tests, and infectious disease tests which commonly employ strip testing using colorimetric, fluorescence, or electrochemical detection methods^5^,^14^. Detection methods employing mobile devices have predominately adapted to the traditional approaches; colorimetric detection has been studied most thoroughly^3^,^4^,^15^,^16^,^17^,^18^,^19^,^20^, though researchers have demonstrated that electrochemical^8^,^21^ and luminescence-based methods^22^ are also feasible options.

Martinez *et al.*^4^ reported the “mobile colorimetric detection” of protein and glucose assays on paper-based microfluidic devices patterned using photoresist; they found that quantifying analyte concentrations using a camera phone to image the test and transfer to a computer for analysis yielded accurate results for medical diagnosis^4^. In some other cases detection has been implemented directly on smartphones using apps developed by researchers for their particular systems to analyze the tests^3^,^15^,^17^,^20^. Wang *et al.*^3^ developed a microchip enzyme-linked immunosorbent assay (ELISA) on poly(methyl methacrylate) (PMMA) substrate for the detection of an ovarian cancer biomarker (HE4) from urine using an integrated mobile app. The built-in camera on the phone was used to image the ELISA and the app was programmed to select the test region and report the pixel values which, when compared to a stored standard curve, was used to determine the HE4 concentration. As well, Delaney *et al.*^23^ established the application of a mobile phone for basic potentiostatic functions through the audio jack which provides the potential for luminophores to generate electrochemical luminescence (detected by the camera subsequently). In the same study, they also developed an app to measure red pixel intensity of the analyte signal against a calibration curve to analyze and display the amount of analyte in the sample^23^. Due to limitations regarding detection and quantitation using solely the smartphone camera, other researchers have turned to the development of devices that clip on to the phone, providing additional detection or imaging capabilities^15^,^16^,^17^,^24^,^25^,^26^. There is no doubt that the amount of interest in furthering this area of research is expected to rise substantially in coming years^27^.

Software development for mobile devices has also grown rapidly in recent years, with readily available public apps ranging from games, news to first aid guidance^28^. One such type of apps that utilizes the phone cameras is the barcode scanner, commonly marketed for price comparisons and product review purposes. The barcode scanners decipher a series of bars and spaces to read the encoded data within. Conceptually different from the bio-barcode assays that utilize nanoparticle probes functionalized with encoding DNA or other specific binding components (i.e., antibodies)^29^,^30^,^31^,^32^,^33^,^34^, herein we describe a barcode-formatted assay protocol that integrates the smartphone camera in reading the assay with standard barcode scanning apps. The objective is to design a *bona fide* barcode assay capable of both qualitative and quantitative analysis with a regular smartphone (no hardware and software driver modifications). In particular, we have developed an assay for the detection of analytes, primarily of biomedical relevance (e.g., protein biomarkers), in which the pattern of the assay binding strips is styled in the manner of a common linear barcode such that it can be detected both visually by the naked eye and by a smartphone barcode scanner. The “data” in the barcode is the qualitative portion of the test, i.e., the presence or absence of the analyte is determined by a barcode scanner app. Quantitation can be then achieved directly on the same assay using a scanometric method with the aid of a self-developed mobile app or computer-based image analysis software.

## Results

### Standard Barcodes and Barcode-formatted Assay Design

**Figure 1:**
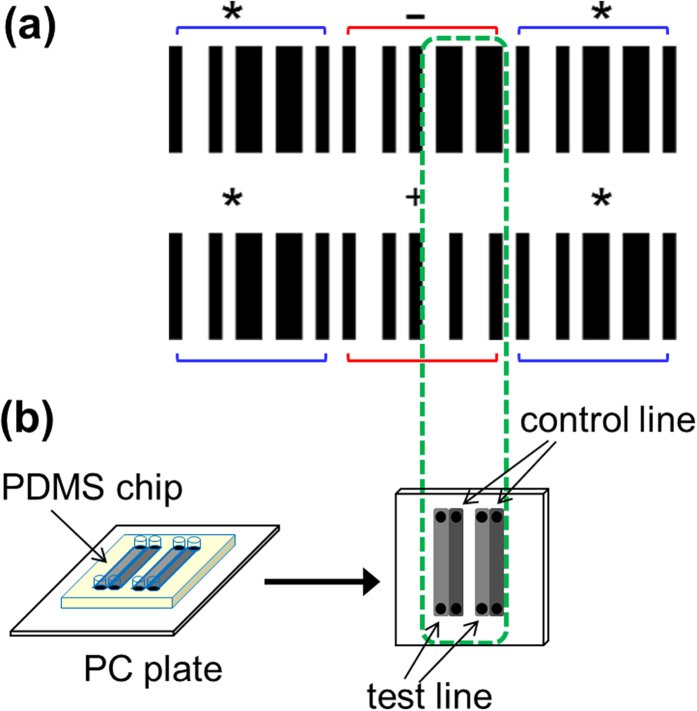
(**a**) Barcodes of “−” and “+” symbols as created using Code 39. The two characters (highlighted with the red brackets above and below the barcodes) are identical apart from the 4 elements in the middle of each character (dashed green box); start and stop characters are denoted by “*” (highlighted with the blue brackets). (**b**) Enlarged image demonstrating the design of the assay strips, as patterned using a microfluidic PDMS chip. The wide bars of the “−” character have been “divided” into four separate binding strips. The first and third serve as test lines while the second and fourth serve as control lines for the test. Barcodes are scanned by placing the PC plate atop a piece of paper with printed barcode, apart from the strips indicated in the green dash-line box. In the case of a positive test, all channels will produce a visible signal that can be scanned, reading as “−”, while in a negative test, only channels two and four will produce a signal, reading as “+”.

As optical representations of data, barcodes consist of a series of bars and spaces, with varying widths, which can be scanned and deciphered by stand-alone readers or scanner apps on smartphones. Each character to be encoded within a barcode has a unique pattern of bars and spaces that is recognizable to the reader or mobile app. The linear barcode is the most common, identifiable to almost everyone as being used in the retail industry for product identification. One such type of linear barcoding system is Code 39 (Fig. 1a), where each character has its own unique pattern consisting of 5 bars and 4 spaces, with three of the 9 elements being wide, and the remaining 6 being narrow. Between each character’s first and last bar is a space, typically narrow so as not to “end” the barcode as it appears to the scanner. At each end of the barcode is a start and stop character (the symbol “*”), which serves as an indicator to the scanner of the reading direction. For the purpose of our assay development, Code 39 is advantageous over other linear systems for our purpose because it offers two characters that have almost identical barcodes, apart from 4 elements, as indicated in Fig. 1a with a green dash-line box. Highlighted with red brackets above and below, the barcodes encoding the “−” and “+” symbols have the same start and stop characters and are identical in the first 5 elements of the code; the differences are the last 4 elements where one has two narrow spaces neighboring two wide bars whereas the other has two wide spaces next to the two narrow bars. Additional technical details on barcodes, specifically Code 39, can be found in the Supplementary Information.

Using this coding system, an assay can be designed to be in the exact format of a barcode and to elicit a color change such that upon binding (or lack of) an analyte, the species of interest, a different barcode symbol will be read. These barcode symbols in our case are the above-mentioned “−” and “+”. Because the rest of the barcode is identical, rather than waste reagents, only the two bars, represented with 4 binding strips (Fig. 1b), are used as the test platform and the remainder printed on white paper using an office printer. A benefit of doing this is that the printed sections can act as a color (or grayscale) reference in the quantitative function of the test. Similar to lateral flow assays, the barcode assay here is conducted only in the test region where each of the two wide bars of “−” are “separated” into two narrow entities (Fig. 1b) in which the first acts as a test line and the second as control line. In the control line, regardless of whether the intended antigen binds, a signal will appear and can be read. The control serves as validity test; the absence of signal indicates that the test is invalid and the result is void. In the test region, a particular antibody is immobilized in the test line to capture the analyte; the presence of analyte will result in a visible signal following addition of the detection antibody and signal enhancement, thus allowing the scanner to read the barcode, provided the test is valid. Therefore, in the case of a positive test where both the test and control lines produce visible signals (represented as a wide bar), the reader will scan the test as “−”. In the case of a valid negative test, “+” will appear due to signals only in the control lines (represented as a narrow bar). The test and control regions can then undergo quantitative analysis where measurements of antigens or analyte can be determined colorimetrically or scanometrically; that is, the intensity of the color or grayscale change as a result of the binding reactions of the second antibody to the analyte and signal enhancement process (silver staining) is compared to the intensity of the background color. This can be accomplished by scanning the samples (using a desktop scanner in the reflectance mode) into the computer as a RGB image for further processing using software (e.g., ImageJ). From the RGB image, the averaged grayscale intensities are found and used to determine the optical density ratio (ODR) given by


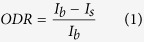


where *I*_*b*_ is the luminosity of the background and *I*_*s*_ is the luminosity of the binding site (the assay strip)^35^.

**Figure 2:**
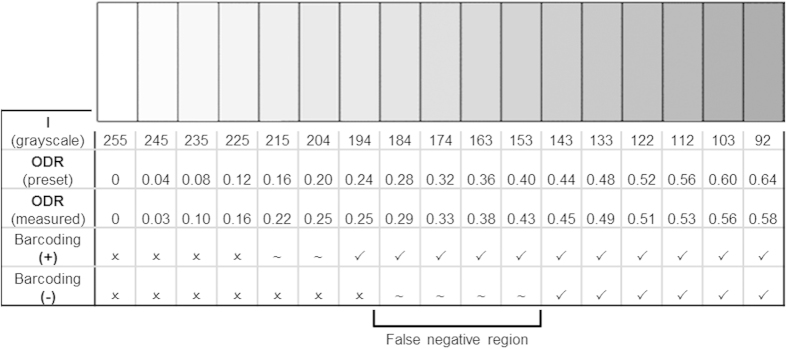
Correlation between the preset grayscale values, the preset ODR, the measured ODR, and the reading capability by the barcode app. The test and control lines (Fig. 1b, the four differential elements) were printed at the preset grayscale values and scanned to determine whether the barcode app could read the result; the remaining bars of the barcode (start, stop, and other elements of the “+” and “−” characters) are printed in black (I = 0).

To determine the detection range that the barcodes can encompass, a range of darkness intensities of barcodes for “−” and “+”, with ODRs from 0–1 (grayscale values between 255 and 0 on an 8-bit grayscale), were printed (Fig. 2) and tested with the mobile app XZing Barcode Scanner^36^ (other free barcode reading apps can also be employed). It was found that when reading “+”, the barcode with an ODR as low as 0.24 can be scanned, while with ODRs of approximately 0.16–0.20, successful barcode reading was intermittent or required longer time focusing and decoding. When trying to read “−”, slightly higher ODRs are required (0.44). Similar to reading “+”, there exists a range (ODRs between 0.28–0.40) where sporadic correct results occurred. As can be interpreted from the data, “−” is more difficult to decipher, creating a region of ODRs below 0.44 that can be considered as the false negative region due to the possibility of a positive assay having a low concentration of analyte that cannot produce a detectable signal by the barcode reading app.

### Proof of Concept: Biotin-Streptavidin Binding Assay Reading

The popularly studied biotin-streptavidin binding assay was used as a model system to evaluate the barcode assay principle because of its well-studied binding interactions; it is known to bind with high affinity (K_a_ ≈ 10^15^ M^−1^) and specificity, thereby making it a suitable model for many bio-recognition interactions^37^,^38^,^39^,^40^.

**Figure 3:**
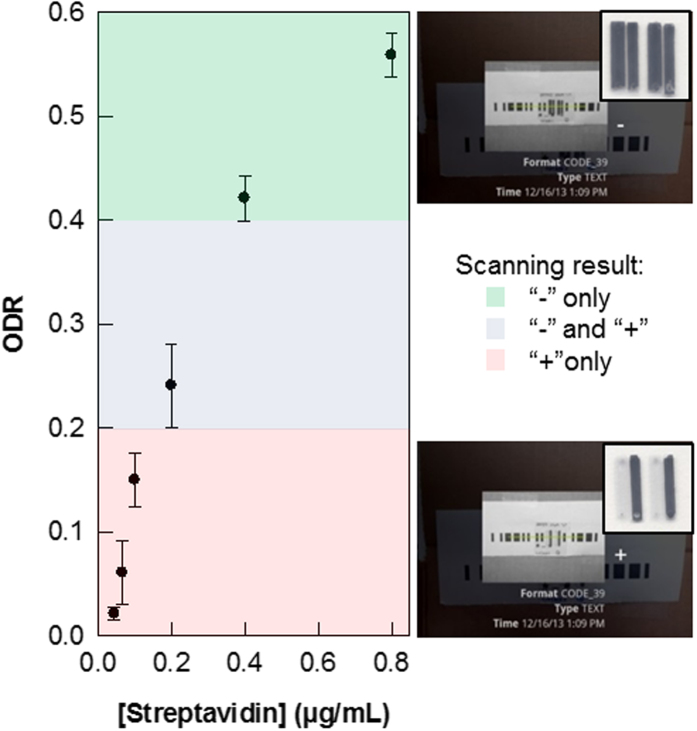
Dependence of ODR on concentration of Nanogold streptavidin conjugate following silver enhancement for biotin-streptavidin assays prepared as “−” and “+” . Scanning by the barcode app is done by placing the PC plate atop a sheet of white paper with the remainder of the barcode printed on it. The different highlighting colors in the plot indicate their feasibility to be scanned by the barcode app: the “−” character can be readily scanned in the green zone, “+”is readable in the pink, and neither is read by the app in the gray zone.

The assay was performed on a polycarbonate (PC) plate using a polydimethylsiloxane (PDMS) chip with embedded microchannels to form the binding strips, i.e., the test and control lines into which the reagents were added (see Methods) to create the barcode pattern shown in Fig. 1(b). The PC plate, after the photo-activation, was first modified with biotin moieties using NH_2_-PEG_2_-biotin (biotin moiety linked to terminal amine by polyethylene glycol spacer) via an amide coupling protocol. Nanogold-streptavidin conjugate was added before silver enhancement (gold nanoparticle-promoted silver deposition) as a means of signal amplification to give easily identifiable signal strips (to be analyzed by ODR). The dependence of ODR on the concentration of nanogold streptavidin conjugate was then determined (Fig. 3). Here, the increasing ODR trend is evident, with the signal reaching saturation between an ODR of 0.50–0.60, which corresponds to high concentrations (0.8 μg/mL) of the conjugate. At concentrations at or above 0.40 μg/mL of the conjugate, both “−” and “+” barcodes can be read by the scanner app. For the “+” barcode, as predicted from previous results, even low concentrations down to 0.20 μg/mL, “+” can be read; concentrations below 0.20 μg/mL can still be analyzed, but cannot be read by the scanner app for its encoded character.

**Figure 4:**
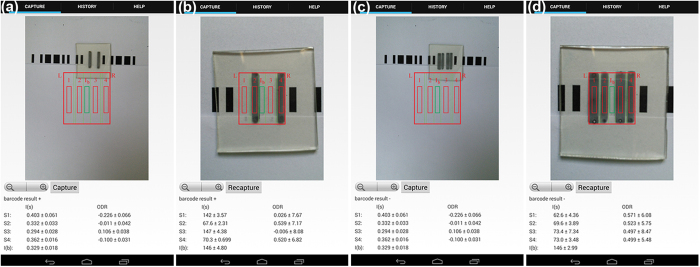
Scanning of the assays with the customized app for (**a**) the encoded barcode result of “+” before (**b**) ODR analysis; and (**c**) the result of the “−” assay and the (**d**) ODR analysis of the four binding strips. The assays presented here are for hCG at 20 mIU/mL.

To improve the general analysis method, particularly the ability to read the barcodes, we have developed an app to scan barcodes and analyze the darkness intensities of the channels (Fig. 4). It is conceptually different from the previously reported mobile app for quantitative scanometric analysis^41^, which was essentially the mobile version of the histogram tool in Photoshop or ImageJ. This customized app is also superior to the free apps because it is capable of obtaining both qualitative and quantitative information. Specifically, the app first scans the barcode for the encoded data, providing live feedback (Fig. 4a,c). The user then positions the channels to obtain the ODR values (Fig. 4b,d and Methods). By encoding the assays, this also introduces the possibility of multiplexing (*vide infra*).

### Real-world Application: hCG Detection and Potential Multiplexing

**Figure 5:**
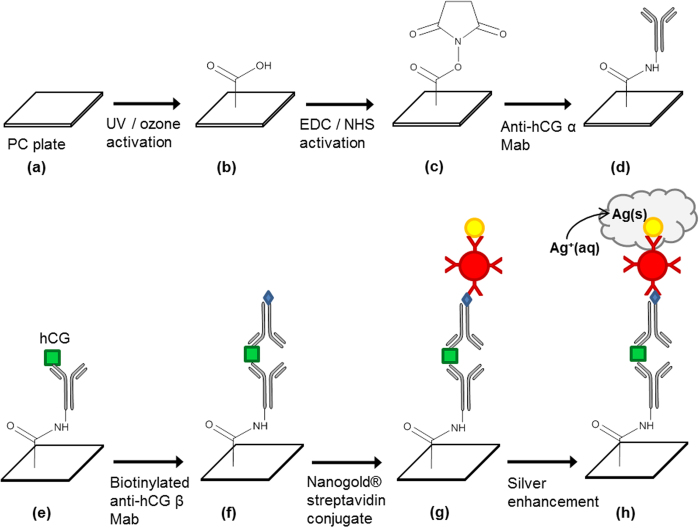
Preparation of the sandwich immunoassay for hCG performed on the polycarbonate (PC) substrate (see Experimental for details). After activating the surface of the PC (**b**,**c**), a monoclonal antibody specific for the α subunit of hCG is immobilized on the surface via an amide-coupling reaction (**d**). The target binds (**e**) and another monoclonal antibody, conjugated to biotin, and specific for the β subunit of the hCG is added (**f**). The Nanogold streptavidin conjugate is added (**g**) before silver enhancement is used to visualize the signal (**h**). In the case of the direct biotin-streptavidin assay, NH_2_-PEG_2_-biotin is added after the surface activation steps; no antibodies are added. Nanogold streptavidin and silver enhancement is also used.

**Figure 6:**
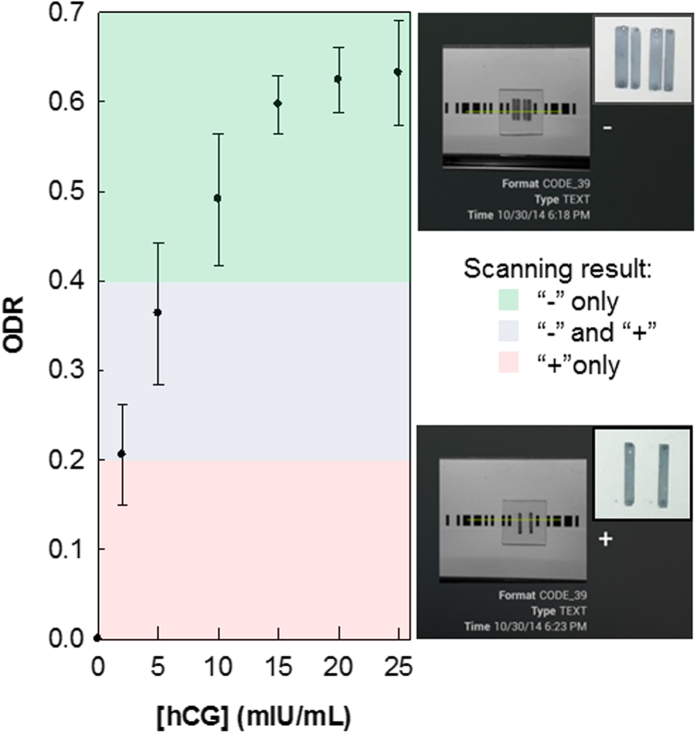
Quantitation of hCG with the barcode assay protocol; ODR as a function of hCG concentration. Tests are performed in triplicate; the errors are the standard deviations of the repeated measurements.

Human chorionic gonadotropin, hCG, is a hormone produced mainly during pregnancy, with low levels present as well in males and non-pregnant women. As pregnancy progresses, hCG levels in the blood and in urine vary substentially. When qualitatively detected, hCG identifies a patient as pregnant, and when concentrations are quantitatively determined, this may also indicate anomalies in the pregnancy^42^,^43^,^44^,^45^. The preparation of the hCG assay on the PC substrate is similar to a standard sandwich-format immunoassay (Fig. 5). The PC surface is activated before a monoclonal (Mab) capture antibody (anti-hCG α Mab) is immobilized. The target is then added (either as a standard solution or urine sample), which binds to the capture antibody. Addition of a biotinylated detection antibody (anti-hCG β Mab) completes the sandwich assay, and as with the biotin-streptavidin assay, nanogold-streptavidin conjugate is added, followed by the gold nanoparticle-promoted silver deposition. As expected, the ODR increases as the concentration of hCG increases, reaching saturation at 15 mIU/mL (Fig. 6). Using the free barcode app, hCG assays as low as 5 mIU for a positive test (“−”) can be scanned, while using the custom app, no limitations apply, i.e. low ODRs provide enough contrast between the bars and spaces to be scanned by the app. Therefore, even concentrations below 2.0 mIU can be detected. At this point however, using the strictly barcode-only app, hCG concentrations ranging from 5 to 25 mIU/mL can be quantitated, which is comparable with the rapid, lateral flow strip tests (“pee on a stick” tests) whose limits of detection are usually between 5.5 and 100 mIU^46^,^47^. Progress is being made to expand the range of detectable hCG concentrations, and to further improve the customized barcode app for reading and analyzing the encoded data in a single scan which shows promise as a quantitation method. This will be validated in our future work, which will involve the test of clinical urine samples from pregnant women.

The specificity of the system has also been tested preliminarily with two hormones related to hCG, namely, thyroid-stimulating hormone (TSH) and follicle-stimulating hormone (FSH). TSH and FSH share an essentially identical α subunit with hCG^48^, but vary in the β subunits. This suggests that while each can bind to the capture antibody, the detection antibody would not bind, and therefore no signal should be observed. The results support this hypothesis as when the two hormones were tested, the obtained ODRs for TSH (0.07 ± 0.02) and FSH (0.05 ± 0.02) were not much different from that of the blank signal (no hCG, 0.04 ± 0.01). TSH and FSH concentrations in the experiments were chosen to be on the high end of the normal range, at 15 μIU and 25 mIU, respectively.

**Figure 7:**
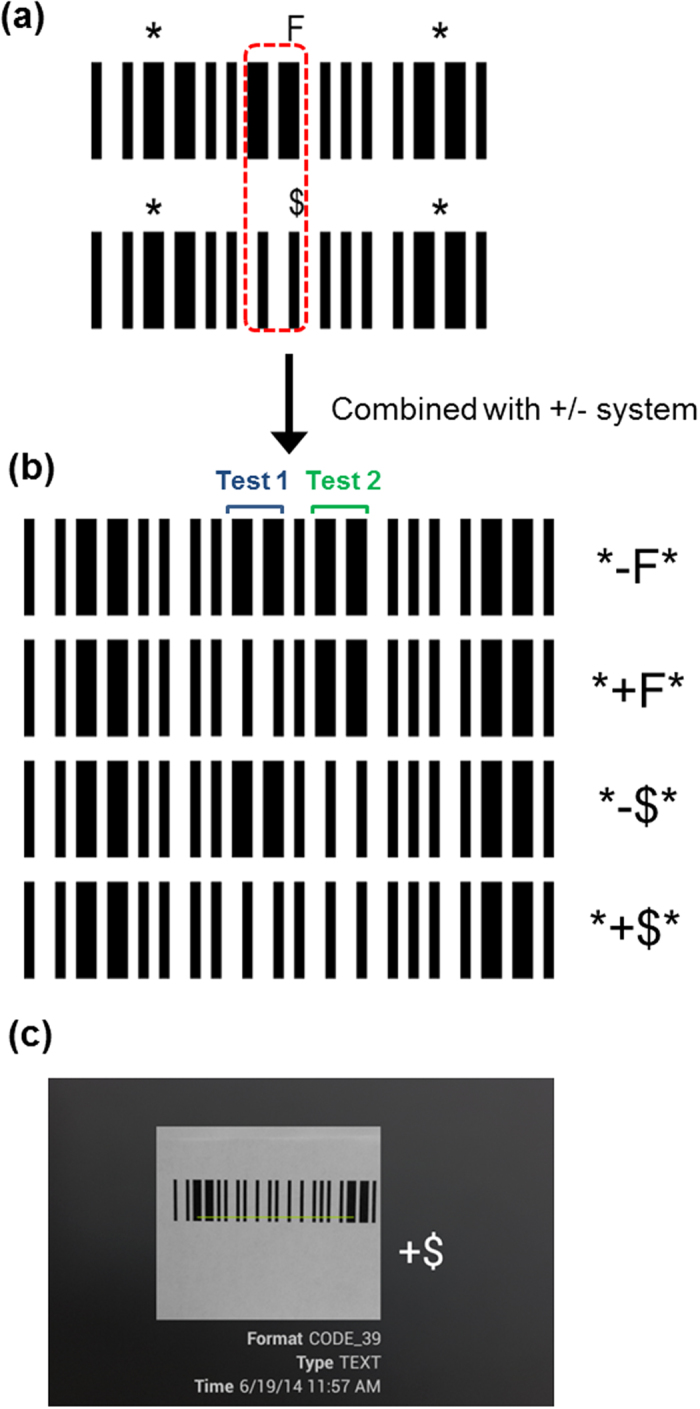
(**a**) Similarities between the “F” and “$” codes. (**b**) Possible combinations of the −/+ and $/F barcodes. One analyte is tested with the +/− (Test 1, blue bracket) characters, another one with the F/$ (Test 2, green bracket) characters. Additional analytes require further extension of the barcode using the same −/+, $/F codes or others. (**c**) Reading of the combined barcode for “+$” with a free app.

Typically, a test for a single marker is considered insufficient or multiple markers can lead to early clinical diagnosis of diseases and disorders since numerous markers are usually indicative of issues^49^,^50^,^51^. For example, a combination of 4 markers, namely hCG, alpha-fetoprotein (AFP), unconjugated estriol (uE_3_), and inhibin A, is currently used to screen for Down Syndrome during pregnancy using sera from the mother^43^,^49^. A multiplexing system is possible for the barcode assay protocol. Due to the nature of Code 39, we can extend the barcode to test for more than one analyte. Apart from the “−” and “+” characters, “$” and “F” also share a similar format (Fig. 7). To multiplex, a test for one analyte is done using the −/+ characters, while another analyte would use the $/F characters. It is possible to test further for an increasing number of analytes with repetition of any of these characters, or addition of different ones with similar formatting.

An alternative multiplexing possibility would be to use two (or more) characters for each analyte in series, whereby the first character (e.g. a letter) would designate the target, while the second character would use the “+/−” system to indicate the presence or absence of the analyte. These pairs of characters would be scanned by the barcode app as “A+”, “B−”, which would allow users to easily determine the results, particularly if a highly multiplexed test was conducted. In that case, users would be able to identify the test result of the n^th^ target simply based on the first character’s designation, without having to track the results of which assay corresponded to the n^th^ test.

## Discussion

The app we have developed can both scan the barcodes for the encoded character and quantify the binding strips after signal enhancement based on the ODR, albeit in two separate steps. We are currently improving the app to be an all-in-one, single scan, qualitative and quantitative platform on a smartphone, as well as tuning the minimum detectable ODR such that the barcode scanning is possible while still maintaining low detection limits. One other current inconvenience is the need for consistent lighting to be provided across all assay strips as any inconsistencies such as glare or preferential lighting (i.e. from left or right sides instead of directly from the top) can lead to errors in the ODR reading. As equation 1 takes into account the intensity of the background, variable lighting is reduced to some extent. An additional option is to implement a lighting-correction algorithm to the app, such as that reported by Hong and Chang^52^. This and other improvements that aid the user (e.g. alignment aids on the PC to help the app self-recognize signal strips) will be implemented in the improved version of the app.

A multiplexing version, is in a preferred form, would allow the user to indicate the number of assays to be analyzed by the app. The current system, however, has first been employed on a PC plate for a single test, but along with the app, other substrates (e.g., paper, glass, and other polymeric materials) are being studied. While the PC barcode assay test now is relatively low cost (approximately $0.20 per test, including all reagents and PC plates), substrates such as paper can be considered more ideal in that they are readily available, inexpensive, disposable, and simpler to work with. With colorimetric tests, the contrast between the white background and the colored portions of the reactions can easily be observed, providing for easier color intensity analysis after imaging with the camera on mobile phone^4^.

Because the assay shown here for hCG is a proof-of-concept, only a single analyte was used. For different targets, even in low multiplexed assays of our barcode sort, it may be simpler to count the number of channels, similar to that of a lateral flow pregnancy test strip. At highly multiplexed assays however, scanning for the encoded data offers a more practical approach. For multiplexed assays, we would able to test a series of related targets (e.g. different pregnancy hormones in urine) and obtain results simultaneously. An advantage of this method, whether they be a single assay or highly multiplexed, is that the app can immediately quantify the amount of target present in the sample based on the ODR, which has been shown previously to be comparable to standard scanometric methods^41^. The scanometric method by desktop scanner and ODR, particularly for hCG, has also been shown, via an alternative disc-based method, to be consistent with the ELISA method^53^. When compared to these, the barcode-smartphone approach is considerably more portable, commonly available, and lower cost (since it does not require any specialized instrumentation such as a plate reader), and has similar assay preparation time as ELISA.

In summary, a smartphone-readable, *bona fide* barcode assay has been demonstrated. The assay was first shown using a direct biotin-streptavidin assay before being applied to the pregnancy hormone, hCG. The linear barcode assay is both qualitative and quantitative when scanned and imaged using a smartphone-app combination, and requires no external accessories, thereby highlighting its potential to boost the research and development of POC devices, particularly by minimizing the need for specialized instrumentation and providing instant testing results on-site.

## Methods

Two characters of Code 39 have been chosen as the primarily used barcodes, namely the “+” and “–” characters (Fig. 1a). Since the barcode is a pattern of bars on a contrasting background, the assay is created using a series of silver-enhanced, dark bars on a white background. Only four channels are used in a single assay, with the remaining bars of the barcode to be printed on a sheet of white paper. Accordingly, a PDMS chip was fabricated to have four microfluidic channels (Fig. 1b) for the test. The first half of the two wide channels have a width of 3 mm, the second half have a width of 2 mm, and the channels are separated by a barrier of approximately 0.5 mm. The PDMS was prepared using the Sylgard 184 Silicone Elastomer Kit (Dow Corning) according to the instructions given; the elastomer base and curing agent were mixed in a 10:1 ratio and applied to the silicon master with features in the dimensions specified above.

### Biotin-streptavidin Assay Preparation

A sheet of PC was cut into the appropriate size (3 cm × 3 cm) and rinsed with ethanol and deionized water before drying with nitrogen gas. The PC plate was placed in a UV/Ozone cleaner for 15 min irradiation and incubated further for 20 min. Activation solution (100 mM EDC (1-ethyl-3-(-3-dimethylaminopropyl) carbodiimide hydrochloride) and 25 mM NHS (N-hydroxysuccinimide) in 0.1 M MES buffer (2-(N-morpholino) ethanesulfonic acid, pH 5.8)) was placed on the surface for 3 h and subsequently rinsed off with deionized water. The surface was dried with nitrogen gas before the PDMS chip was attached to the surface. NH_2_-PEG_2_-biotin solution (30 μM) was pipetted into the channels and allowed to remain for an hour. The channels were rinsed with a 100 mM phosphate buffer solution (containing 500 mM NaCl, 0.8% bovine serum albumin (BSA), 0.1% gelatin, 0.05% Tween 20, and 2 mM NaN_3_ at pH 7.4) for 15 min. To prevent non-specific adsorption, the surface was passivated with an 8% BSA solution for 2 h. The channels were then rinsed again with the phosphate buffer for 15 min before a nanogold streptavidin conjugate solution (0.04–0.8 μg/mL) was injected and incubated in the channels for 50 min before rinsing with the phosphate buffer solution. The PDMS chip was removed and the PC surface was washed with deionized water and dried with nitrogen gas. The reaction area was then subjected to silver enhancement with a solution of 0.024 M silver nitrate and 0.091 M hydroquinone and 4% gelatin in citrate buffer (pH 5.4–5.6) for 10 min (with very low signal beginning to appear within 4 min). The PC plate was then rinsed once more with deionized water to stop the reaction and dried before being read and analyzed.

### hCG Assay Preparation

A schematic representation of the assay is shown in Fig. 5. As with the previous method, the PC plate was first photo-activated, and treated with the EDC/NHS solution. The surface was then rinsed with deionized water and dried with N_2_ gas before the PDMS chip was adhered to the surface. Into each of the microfluidic channels anti-αhCG antibody (50 μg/mL, in 10 mM phosphate buffer with 150 mM NaCl, 5% glycerol, at pH 7.4) was added and allowed to immobilize on the surface for an hour. The surface was then passivated with 10 mM PBS buffer containing 2% BSA and 0.5% Tween 20 at pH 6.0 for 2 h to prevent non-specific binding. Channels were then rinsed with 10 mM PBS buffer (with 0.5% Tween 20); the introduction of a hCG-containing solution to the channels (incubation period of an hour at room temperature, diluted with 10 mM PBS buffer) would allow binding between hCG and the antibody immobilized on the surface. The second antibody required in the sandwich assay is biotinylated anti-βhCG (0.1 μg/mL) which was added to the channels of the PDMS and remained for one hour at room temperature before rinsing. Nanogold streptavidin conjugates (0.8 μg/mL) were then added and incubated for 50 min, before the silver staining treatment. The same concentrations of solutions and staining time are used for the hCG assay as the biotin-streptavidin assay (the stain begins to appear between 5–6 min in this case). The signal detection and analysis were also performed in a same way as the biotin-streptavidin assay using the same smartphone app and/or scanner. The concentration of the analyte was determined by obtaining an image of the barcode and an analysis of the optical density ratio relative to that of a standard curve. The reagent concentrations and reaction times have not been tested thoroughly; shorter time for a test can be certainly achieved through optimization of all experimental conditions.

### Qualitative and Quantitative Analysis

The PC plates with stained binding strips were positioned on top of a piece of white paper with printed partial barcodes (the remaining bars of the barcode as seen in Figs 3, 4 and 6) printed and scanned using a freely available barcode scanner^36^ installed on a Blackberry Q10, Samsung Galaxy S2, and a Nexus 7 tablet, situated 10 cm from the sample. In the second part, the samples were scanned into a computer using a desktop scanner (reflectance) and using Image J, the averaged grayscale intensity of the test lines, control lines, and the background were determined. Using these values, the ODRs were calculated using Eq. 1.

For the quantitative tests, a custom app was written using the Android Developer Tools in Java and installed on a Google Nexus 7 device running Android version 4.4.3. The app uses a weighted average grayscale (luminosity) based on the RGB color mode^41^,


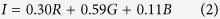


Launching the app opens the rear camera of the mobile device and the screen displays a preview from the camera (Fig. 4 and Supplementary Information video). For the purposes of the video, the sample is subject to ambient lighting, but for a typical ODR measurement, samples are placed 7 cm directly under the phone inside a dark containment with a single light source (40 W) to give even lighting across all bars in the sample. The sample is to be positioned into the centre of the screen: the red bars indicate the signal regions, while the green bar indicates the background region, into which the user aligns using the zoom button or manual positioning of the phone. Capturing the image commands the app to obtain the raw CMOS data from the camera and to convert each color pixel into RGB values, thereby giving an array of RGB values. For each pixel, the *I* value is calculated as above; the app samples three groups of pixels in each of the test strips (*I*_*s*_) and background (*I*_*b*_) (each group has 4715 pixels) and the average and standard deviation of the *I* is determined. Using the mean grayscale value of the background, the ODR value for each test strip is obtained by using Eq. (1), which was described in the results section. The intensities of the signal and the calculated ODRs, along with the barcode results, are then displayed to the user.

## Additional Information

**How to cite this article**: Wong, J. X. H. *et al.* Direct Reading of *Bona Fide* Barcode Assays for Diagnostics with Smartphone Apps. *Sci. Rep.* **5**, 11727; doi: 10.1038/srep11727 (2015).

## Supplementary Material

Supplementary Movie 1

Supplementary Information
