# Supplementary material for: Direct Reading of Bona Fide Barcode Assays for Diagnostics with Smartphone Apps
Source: Sci Rep. 2015 Jun 30;5:11727. doi: 10.1038/srep11727 (PMC4485167; doi:10.1038/srep11727)
Supplement: Supplementary Information [file srep11727-s2.pdf]

Supplementary Information

Direct Reading of *Bona Fide* Barcode Assays for Diagnostics with Smartphone Apps

Jessica X. H. Wong,[†] Xiaochun Li,[‡,*] Frank S. F. Liu,[†] and Hua-Zhong Yu[†,‡,*]

[†] *Department of Chemistry and [‡]Department of Molecular Biology and Biochemistry, Simon Fraser University, 8888 University Drive, Burnaby, British Columbia V5A 1S6, Canada*

[‡]*Key Laboratory of Advanced Transducers and Intelligent Control Systems (Ministry of Education), College of Physics and Optoelectronics, Taiyuan University of Technology, Shanxi 030024, P.R. China*

** Corresponding Author: E-mail: hogan_yu@sfu.ca (H.Y.); lixiaochun@tyut.edu.cn (X.L.)*

1. Standard barcodes and barcode-formatted assay design

Barcodes are optical representations of data; they consist of a series of bars and spaces, with varying widths, which can be scanned and deciphered by readers or scanners. Each character to be encoded within a barcode has a unique pattern of bars and spaces that is recognizable to the reader. Linear barcodes are available in many different types; in retail, the most commonly used is the U.P.C. (Universal Product Code), libraries prefer to use the Interleaved 2 of 5 system, and the E.A.N. 8 or 13 systems that is used in both retail and on books. Some barcode systems have yet to find widespread public usage, such as Code 39. Different elements are incorporated into different types of barcodes; some are limited to encoding of numbers, others are able to encode more characters, while some can encode more information within a particular area than other types. There are two formats of encoding data into linear barcodes. In one method, the encoding of data is done so continuously; in other words, the pattern of each character in the barcode begins with a bar and ends with a space, with the next character's pattern beginning immediately after the last character's space. A discrete barcode on the other hand, begins and ends with a bar. A space is an added element used to separate between

the last bar of one character and the first bar of the next, and is ignored by the scanner if it can read this particular barcode type. In order to encode the numerous possibility of characters (e.g., letters, numbers and special characters), barcodes, as previously mentioned, operate under a system of variable bars and widths; the patterns are not such that one letter has a single bar and another letter has two bars. Each character in a single barcode type have the same number of elements; for example, Code 39 (or Code 3 of 9) characters all have 5 bars and 4 spaces (9 elements, 3 of which are wide elements) that make up the code and it is the variation in the width of the bars and spaces in a particular pattern that codes for a character. The variation in patterns between characters allows for the design of a test such that depending on the result of the test, a different character can be generated.

Code 39 demonstrates a discrete barcode system where the widths of the bars and spaces are made of only two types, narrow or wide. It can encode only the upper case letters (A-Z) of the Latin alphabet and the Arabic digits (0-9), in addition to 7 special characters (space, -, +, ., $, /, %). As previously mentioned, each character has its own unique pattern consisting of 5 bars and 4 spaces, with three of the nine elements being wide, and the remaining six being narrow. The concept of wide and narrow is defined by the relative widths of the bars and spaces to other bars and spaces; the ratio can be set between 2:1 and 3:1. Between each character's first and last bars is a space, typically narrow so as to not "end" the barcode as it appears to the scanner. At each end of the barcode is a start and stop character (the symbol "*"), which is used as an indicator to the scanner of the direction of reading. A disadvantage of the 39 coding system is that it requires a greater area to encode a particular set of data compared to some of the previously mentioned linear coding types. As well, the system can be considered simplified as it does not employ a check character (that changes depending on the data encoded) as a method of checking the barcode in the event of the introduction of error during the barcode's transmission or storage. Although appearing to be slightly disadvantageous in this manner, the coding system still allows for a checking mechanism in that single misprint of a bar or space will not result in an erroneous reading because it would not encode for any characters; the scanner will act as if no barcode is present.

Code 39 does have an advantage over some other linear systems, e.g., it offers two characters having almost identical barcodes, apart from 4 elements, as a result of not employing an individual checking character. The barcode encoding for the "-" and "+" symbols have the

same start and stop characters, and are identical in the first 5 elements of the code; the differences are the last 4 elements where one has two narrow spaces neighboring two wide bars whereas the other has two wide spaces next to between the two narrow bars (Figure 1a of the main text).

2. Preparation of biotin-streptavidin and hCG immunoassays on PC

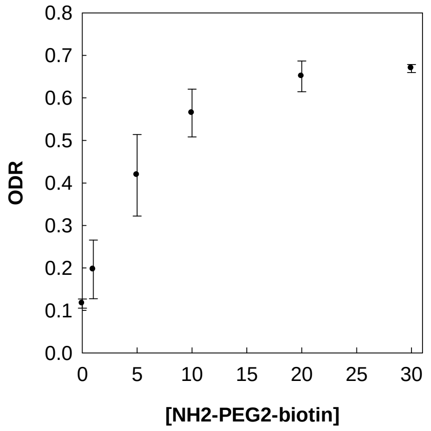

Figure S1– ODR as a function of the NH_2-PEG_2- biotin concentration in the initial test of biotin-streptavidin assay. With the same concentration of Nanogold-streptavidin conjugates, it is clear that the binding signal (ODR) rises initially and then saturates at a concentration of 20 µg/mL.

3. Barcode assay reading

Video S1 - Demonstration of the custom app, i.e., reading the barcode assay and analyzing the ODR of the binding strips.
